# Exploring the Impact of Personal Factors on Residents’ Willingness to Undergo Primary Care Initial Diagnosis in Beijing, China: A Mixed Methods Research

**DOI:** 10.3390/healthcare12232451

**Published:** 2024-12-05

**Authors:** Yongchuang Gao, Yuangeng Guo, Zhennan Wu, Wenhao Deng

**Affiliations:** 1 School of Labor and Human Resources, Renmin University of China, 59 Zhongguancun Street, Beijing 100872, China; gyc5896@163.com (Y.G.); wuzn@ruc.edu.cn (Z.W.); 2 School of Economics and Management, Tsinghua University, 30 Shuangqing Street, Beijing 100084, China; gyg21@mails.tsinghua.edu.cn; 3 School of Management, Beijing Institute of Technology, 5 Zhongguancun South Street, Beijing 100081, China

**Keywords:** primary care initial diagnosis, community health service center, chronic disease management, medical seeking behavior, primary healthcare system

## Abstract

**Background**: As the foundation and core strength of the healthcare system, the primary care initial diagnosis system has been receiving attention from both the medical and management communities. This study aimed to analyze residents’ attitudes toward the system and explore the influencing factors relating to the system in Beijing; **Methods**: Different methods were used to analyze the multidimensional data. This study selected 610 Beijing residents to complete the survey and used a purposeful sampling method to recruit 15 participants aged 25 to 70 for face-to-face individual semi-structured interviews, using both quantitative and qualitative research methods to carry out statistical analysis. **Results**: The tie-breaking age of the interviewees was 46 years old, and the questionnaire showed that highly educated and aging populations had low willingness to undergo primary care initial diagnosis, 97.7% of chronic disease patients were unwilling to undergo primary care initial diagnosis, and different people have different levels of willingness to undergo primary care initial diagnosis. Factors such as level of confidence in the government and health literacy significantly affected residents’ willingness. **Conclusions**: This study suggests that the government needs to foster a positive policy image and actively publicize policy content and effects to increase the confidence of the population in the government. Community health service centers need to use Internet technology to perform chronic disease archiving and management efficiently in order to solve the problems of chronic disease management. The government and hospitals need to focus on the characteristics of different groups of residents and enhance their health literacy so as to implement the primary care initial diagnosis policy.

## 1. Introduction

Primary healthcare systems constitute a crucial component of global healthcare infrastructures, and many countries have well-developed primary healthcare systems [1,2,3], e.g., the United Kingdom and Denmark have implemented well-established family doctor system [4,5], the gatekeeper system in the United Kingdom and the Netherlands emphasizes the importance of primary care physicians, and health services in these countries are better than elsewhere [6]. However, in some developing countries, such as China, the primary healthcare system remains nascent, necessitating urgent enhancement measures [7,8,9,10]. In addition to this, external environmental shocks have also hindered the development of the healthcare system. On the one hand, the increasing aging of the population, with the number of people over 65 years of age globally expected to reach 1.5 billion in 2050 [11], is a major challenge worldwide, and there is an urgent need for governments to address the healthcare needs of this aging population. Additionally, the inequitable distribution of healthcare resources impairs the accessibility and equity of services. Evidence shows that 82% of healthcare resources in China are concentrated in tertiary and secondary hospitals [9,12], resulting in a large number of patients not being able to receive effective treatment in community health service centers. The World Health Organization has advocated for a fortified primary healthcare system to enhance service provision [12,13]. At the same time, studies have shown that primary care health service, as gatekeeper to the health service system, has a significant impact on its quality and level [4,6].

Primary care initial diagnosis is one of the cornerstones and key systems within a hierarchical medical policy that aims to guide patients by giving priority to primary healthcare institutions [3,14]. On the one hand, community health service centers are usually located near patients who can receive medical services quickly, and primary care initial diagnosis can determine whether follow-up treatment is needed. On the other hand, the primary care initial diagnosis policy works in synergy with other policies, e.g., the referral policy can be used to lead to better utilization of hospital resources [15]. China is working to provide a basic medical and health system as a public good to the entire population in order to efficiently implement its primary care initial diagnosis policy and to strive for the modernization of health care. At the same time, the Internet health program uses modern information technology to promote the construction of big medical data to serve policy implementation. Community health service centers, as primary healthcare institutions in China’s public hospital system, are the foundation and core in providing medical services and solving medical problems [1,12], but under-utilization of community health services centers is an important issue of concern [16,17]. At the same time, due to the special characteristics of China’s national conditions, extensive administrative fragmentation has made different organizations responsible for different policy functions, and such a phenomenon leads to inefficiencies in management and difficulties in policy implementation [15]. The implementation of the primary care initial diagnosis system can, firstly, improve the continuity of medical care and the accessibility of medical resources [18] and facilitate access to medical care for the aging and chronically ill groups. Secondly, the higher reimbursement rate for consultation at community health service centers can effectively solve the dilemma expressed as “it’s difficult and expensive to see a doctor” [19,20,21] and improve the fair delivery of medical services. Thirdly, the grading and triage of patients can alleviate the medical pressure on high-level hospitals [3,12,22], promote the construction of hospital unions, and make contributions to the success of China’s medical reform.

Starting in 2015, different provinces and cities in China began to pilot the implementation of this hierarchical medical policy, with some cities achieving limited success while others are still in the exploratory stage [8,23]. The primary care initial diagnosis system is an important foundation for achieving the goal of implementing a hierarchical medical policy in China [24,25], and as the 10th anniversary of its implementation approaches, it is necessary to analyze residents’ attitudes to the system and the factors that influence it and make further improvements to the policy. Furthermore, there are some gaps between present research and the reform progress and practical needs. Studies have found that factors such as the distance to the township healthcare center, the level of medical equipment, and single-visit price acceptance can significantly influence patients’ willingness to attend for primary care initial diagnosis [16,17,18,26]. Some studies showed that residents are more interested in factors such as the medical level of doctors and whether or not they themselves have employee health insurance, which can influence their willingness [27,28]. Other work examined the importance of factors such as age and level of education on the willingness to attend primary care for an initial diagnosis [12]. Moreover, studies in recent years have shown that the government plays an important role in residents’ willingness to go for primary care initial diagnosis and that factors such as the government’s control over policies and the government’s credibility can significantly change residents’ health care behavior [18,29]. Other studies have confirmed the importance of residents’ personal factors, not only demographic variables but also factors such as residents’ health awareness [12,30]. Nevertheless, although many studies have analyzed the factors influencing residents’ willingness to attend primary care initial diagnosis, there are even more influencing factors that need to be explored in depth [12,16,17,18,26,27,28]. In particular, compared to objective factors, such as the distance of hospitals, the medical level of doctors, and the cost of consultation, personal factors, such as residents’ own health status and the level of trust in Internet medical care, also need to be focused on [29,30].

To sum up, this study focuses on Beijing residents as the research subjects to examine the attitude towards primary care initial diagnosis and to identify overlooked influencing factors through quantitative and qualitative research methods, thereby enriching findings in this research area. Specifically, the quantitative study aimed to assess Beijing residents’ attitudes toward primary care and identify potential influencing factors. The qualitative research was conducted to validate the findings of the quantitative study and uncover additional insights not captured by it. This study aims to contribute to addressing the uneven allocation of healthcare resources, promoting the accessibility of healthcare services, and offering policy recommendations applicable to other regions and countries.

## 2. Materials and Methods

### 2.1. Study Design

This study adopted a mixed research design combining quantitative and qualitative research methods. In the quantitative research design, this study employed an internationally recognized scale and a self-developed questionnaire on primary care initial diagnosis based on previous research. The questionnaire gathered data on the willingness of Beijing residents to receive primary care initial diagnosis. The quantitative research design complies with the requirements of STROBE [31]. In the qualitative research design, this study conducted semi-structured interviews with participants to gather data on Beijing residents’ perceptions of and suggestions for primary care initial diagnosis. The qualitative research design meets the COREQ standards [32].

Regarding the questionnaire data, considering the rapid development of the Internet, more and more scholars are using online platforms for conducting questionnaire surveys and collecting data. This study was based on a questionnaire collected through Credamo, an internationally recognized platform for questionnaire collection [33]. Firstly, a credit score of 80 or above was set for participants, as a higher credit score indicates higher questionnaire quality. Secondly, the historical adoption rate of participants was set to 80% or higher to avoid low-quality questionnaires due to unfamiliarity with the answering process. Thirdly, only one participant within 1 km was allowed to avoid sample homogeneity caused by participant clustering. Finally, two preset questions were included: “Do you know about the primary care initial diagnosis policy?” and “Have you been treated in public hospitals such as community health service center in Beijing in the past 90 days?”. The questionnaire survey could only be conducted if both questions were answered “yes”. Meanwhile, the question “Have you learned about the primary care initial diagnosis policy through this questionnaire?” was included in the questionnaire to screen out participants already familiar with the primary care initial diagnosis policy.

### 2.2. Participants

The questionnaire survey was officially released online on 25 June 2024. It was expected that 600 valid questionnaires would be collected. The first round of the questionnaire survey was completed on 10 July 2024. A total of 620 questionnaires were collected. They were eliminated using a system (e.g., the response time was too short, within 5 min) and manual elimination (e.g., knowing about the policy through this questionnaire), and 477 valid questionnaires were obtained. The second round was completed on 21 July 2024, and 105 supplementary questionnaires were collected. Through the system and manual double-elimination process again, 95 valid questionnaires were obtained. The third round ended on 5 August 2024, and 38 valid questionnaires were obtained. At this point, the online questionnaire survey process ended, and 610 valid questionnaires were finally recovered. The 610 valid questionnaires in the Beijing area can better support the purpose of the study, address the research problems, and achieve the expected goals.

For the interview data, we used a purposeful sampling method to recruit participants aged 25 to 70 years for individual interviews during the period from February to March and August to September 2024, with an average age of 46 years. By the time the interview reached the 15th participant, we had enough usable information, and no new insights emerged from subsequent interviews, so we ended the process. These 15 participants came from different districts of Beijing and had experience visiting public hospitals within the past 30 days. They had varying degrees of understanding and participation in the primary care initial diagnosis policy and were expected to provide useful and specific information. The interviews were semi-structured and conducted face-to-face. When contacting participants, we explained the purpose and general content of the interview and informed them that the interviews would be recorded. The interview began only after obtaining participants’ consent. All interviewees gave verbal consent to be interviewed and agreed that the content of the interviews would be used for academic research. The duration of most of the interviews was 20 to 30 min, and information was collected on the effects, challenges, trends, and influencing factors in the primary care initial diagnosis policy from the patients’ perspective.

### 2.3. Data Collection Tools

For the quantitative study, the measurement scales used were derived from two sources. One source was a self-developed questionnaire based on previous research, and the other was a well-established, internationally recognized scale. The contents of the formal questionnaire in this study include the following: (1) Awareness, recognition, and satisfaction with the policy. (2) Selection of hospitals according to disease type. (3) Personal health status, health literacy, E-Health literacy, and level of trust in Internet medical care. (4) The importance of factors influencing the willingness for primary care initial diagnosis. (5) Demographic variables.

The awareness, recognition, and satisfaction with primary care initial diagnosis policy in Beijing question was based on the research by Li et al. [34] and involved asking participants to rate their degree of the awareness, recognition, and satisfaction of the participants. Participants rated these items from 1 to 5 points according to their actual situation. The higher the score, the higher the degree.

Selection of hospitals based on disease type: When conducting the questionnaire survey in this study, we first classified the diseases, including common disease, acute disease, chronic disease, severe disease, and so on, providing reference examples for each type. After that, used the classification of Beijing public hospitals, tertiary hospitals, secondary hospitals, primary hospitals, and community health service centers. Participants were asked to answer according to their actual situation and willingness.

Personal health status was measured using a 3-item scale [35], with an example item such as “My health is excellent”. Health literacy and E-Health literacy were measured using a 6-item scale [36] and an 8-item scale [37], including items such as “Can you use the doctor’s advice (including treatment or behavior) to make decisions about your condition?” and “I know how to use the internet to answer health questions”. The level of trust in Internet medical care was measured using a 3-item scale, with an example item of “I think it’s desirable to submit health information on the web”. These variables were measured on a 5-point Likert scale, with higher scores indicating greater levels of the variables.

The importance of factors influencing the willingness for primary care initial diagnosis: In addition to the above factors related to residents’ own condition, and based on previous literature [38,39,40] as well as comprehensive considerations, such as literature mining, expert interviews, and practical inspections, we added the “level of confidence in the government” and other factors not studied in previous literature. The options were divided into “very unimportant”, “less important”, “general”, “more important”, and “very important” and were assigned a score from 1 to 5 points.

Demographic variables included gender, age, education level, insurance type, and occupation.

Regarding the acquisition of interview data, the interview outline was designed in accordance with previous studies [39,40]. The main questions included “Are you aware of the primary care initial diagnosis?”, “Are you satisfied with the primary care initial diagnosis policy publicized by the government and hospitals?”, “What factors do you think will influence your choice of medical treatment?”, “In the current healthcare reform, what suggestions do you have for the policy content of the government and hospitals?”, “Do you usually exercise? How often do you do so?”, “How do you feel about your own health literacy in terms of seeking medical treatment?”, “Do you seek medical treatment online?”, and “What type of health information do you obtain online?”.

### 2.4. Data Analysis

After eliminating invalid response data, this study used SPSS 25.0 to perform statistical analysis on the valid data. The statistical methods used included descriptive statistical analysis, univariate ANOVA analysis, and multiple linear regression analysis. Firstly, we described the basic information and attitudes of participants toward the primary care initial diagnosis policy. Secondly, we used the exploratory function to conduct a normality test of the data, which was normally distributed and met all regression conditions (*p* > 0.05). Thirdly, after the normality test, data could be used for ANOVA and independent samples T test. We then conducted the univariate ANOVA and independent samples T test. According to classification criteria [24], we classified the participants into different groups based on gender, age, education level, occupation, and so on. Among them, age was divided into ten-year intervals, education level ranged from high school and below to postgraduate education, insurance type was categorized according to common types in China, and occupation broadly covered all types in the market, allowing us to identify differences in willingness for primary care initial diagnosis among different population. Finally, the data met all requirements for regression analysis beyond normality. We used multiple linear regression analysis to identify the relationship between different factors and willingness for primary care initial diagnosis. The above analysis allowed us to identify key factors affecting willingness for primary care initial diagnosis, enabling us to make policy recommendations based on these factors.

**Figure 1:**
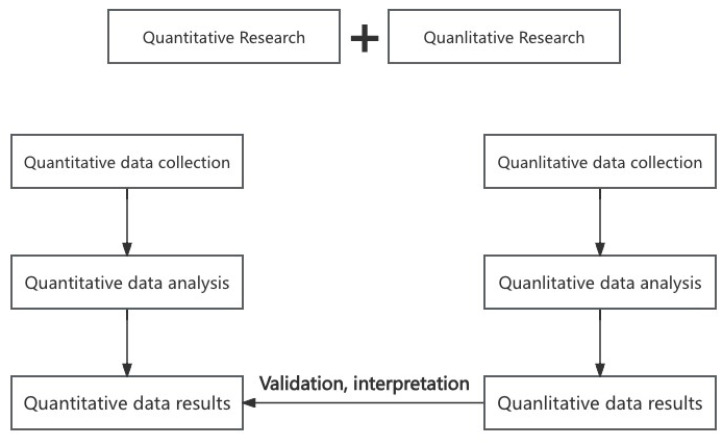
Phases of mixed methods research and data integration.

In addition to the quantitative analysis, we conducted a simple qualitative analysis using an audio recording, transcription, and examination approach. Four researchers were involved in the qualitative study, two of whom were responsible for questioning interviewees and obtaining information from the interviews according to the interview outline. The other two researchers were responsible for the follow-up tasks. During the interview, all content was digitally recorded, transcribed verbatim, and translated into English for analysis, while the main points of the interview were noted. Later, one student assistant transcribed all interviews, and another verified the accuracy of the transcripts. After that, the interview data, on the one hand, verified the results of the quantitative analysis. On the other hand, the content of the interviews provided more background information and details, offering additional insights to explain the statistical trends. Figure 1 illustrates the phases of mixed methods research and data integration. This study included both quantitative and qualitative research designs, with a concurrent explanatory design being used. Quantitative and qualitative data were collected in different ways and at nearly the same time. After data collection, the two different types of data were processed and analyzed, Finally, the results of the qualitative data validated and interpreted the quantitative results.

## 3. Results

### 3.1. Basic Information of Residents

In this survey, Table 1 shows that the proportion of male and female residents is equal, each accounting for about 50%. In terms of age, residents aged 31 to 40 are the most numerous, totaling 334 individuals (54.8%), followed by residents aged 21 to 30 and 41 to 50, each accounting for about 20%. In terms of education level, individuals with higher education accounted for a larger proportion. Among them, the largest group was undergraduates, 383 individuals (62.8%), while those with high school education and below were fewer, totaling 59 people (9.7%). In terms of insurance type, the majority have basic medical insurance for urban employees (459 individuals, 75.2%) and urban residents (74 individuals, 12.1%). In terms of occupation, employees in enterprises and institutions accounted for the largest proportion, totaling 504 individuals (82.6%), with other occupational groups being smaller.

### 3.2. Residents’ Attitudes Toward Primary Care Initial Diagnosis Policy

The survey results showed that the residents’ average awareness of the primary care initial diagnosis policy is 4.20 (SD = 0.700), the average recognition is 4.24 (SD = 0.734), and the average satisfaction is 4.30 (SD = 0.784). It can be seen in the above data that the residents have high levels of awareness, recognition, and satisfaction with the primary care initial diagnosis policy, with awareness being the lowest among the three and satisfaction being higher than recognition.

### 3.3. Residents Choose Hospital Level According to the Disease Type

This study investigated which level of hospital residents would choose for medical treatment if they suffered from a certain disease. The results in Table 2 showed that when residents suffer from common diseases (such as cold or fever) or need recovery after illness (such as massage or traction), the vast majority of people choose community health service centers and primary hospitals for treatment, of whom 493 patients (80.8%) with common diseases prioritize community health service centers for diagnosis and treatment, while very few choose secondary or tertiary hospitals. For patients with acute diseases (such as appendicitis or acute gastroenteritis), most choose secondary hospitals, accounting for about 45%. For patients with chronic diseases (such as diabetes, hypertension, or chronic stomach problems), the majority (405 people, 66.4%) choose secondary hospitals as their first choice, and some patients also go to primary hospitals, but only a very small number of patients with chronic diseases (14 people, 2.3%) prefer community health service centers. In addition, the vast majority of patients with severe diseases (such as tumors or uremia) (512 people, 83.9%) and those with rare or complex diseases (such as acromegaly, hemophilia, or ALS) (502 people, 82.3%) choose tertiary hospitals for treatment, while very few such patients choose community health service centers and primary hospitals.

### 3.4. Factor Analysis of Residents’ Willingness Regarding Primary Care Initial Diagnosis

Studies have shown that public satisfaction is a better predictor and representative of their willingness to participate and perform [41,42,43,44]; therefore, this study used the level of satisfaction with the primary care initial diagnosis policy to represent the level of residents’ willingness to participate in primary care initial diagnosis. The effects of demographic variables, including gender, age, and education level, on willingness to participate in primary care initial diagnosis were explored. The results indicated that gender, age, and education level significantly affected residents’ willingness to participate in primary care initial diagnosis, with statistically significant differences (Table 3). Among them, men exhibited significantly greater willingness than women, with a score of 4.48 (*p* < 0.001). Regarding age (*p* < 0.001), residents aged 41–50 showed the greatest willingness to participate in the primary care initial diagnosis policy (4.45), while those aged 51–60 had the least willingness (3.71). Regarding education level (*p* < 0.001), the higher the education level from high school to undergraduate, the greater the willingness of residents to participate in the policy. However, residents with postgraduate education exhibited significantly reduced willingness, scoring only 3.79. Regarding the type of insurance, willingness to seek primary care initial diagnosis is higher for those with basic medical insurance for urban workers and urban residents, while it is lower among those with commercial or publicly funded medical insurance.

To further analyze the factors influencing residents’ willingness to participate in primary care initial diagnosis, this study conducted a multiple linear regression analysis. The results showed that the adjusted R^2^ is 0.433 (*p* < 0.001), indicating that these factors explain 43.3% of the change of variance in residents’ willingness to participate in primary care initial diagnosis. The results indicated that the level of confidence in the government, personal health status, health literacy, and trust in Internet medical care significantly affect residents’ willingness to participate in primary care initial diagnosis. Based on the data analysis in Table 4, this study draws the following conclusions: residents are more willing to participate in primary care initial diagnosis when they have greater confidence in the government, residents are more willing to participate in the policy when they have better health status, residents’ health literacy significantly increases their willingness to participate in primary care initial diagnosis, and they are more likely to choose community health service centers when they trust the Internet as a source for seeking medical treatment.

### 3.5. Residents’ Views on Primary Care Initial Diagnosis

In this study, 15 Beijing residents were interviewed to understand their views on primary care initial diagnosis. Table 5 presents some of their views and opinions on primary care initial diagnosis. Based on the results in Table 5, we can see that this interview included participants from various age groups and backgrounds, including highly educated individuals and those with chronic illnesses). According to the interview transcripts, the elderly population feels that primary care initial diagnosis does not facilitate their consultation process, while the highly educated individuals prefer to visit tertiary hospitals, as they are less familiar with community health service centers. Individuals with chronic diseases, as the key target of the primary care initial diagnosis policy, sometimes refuse to participate in primary care initial diagnosis due to the shortage of medication and the cumbersome treatment process. At the same time, some respondents expressed strong confidence in the primary care policy and mentioned their trust in the government’s healthcare sector. Additionally, several interviewees stated that they would use the Internet for medical consultation, as they found it more convenient and faster, allowing them to consult doctors and purchase the necessary medications. Regarding their personal situation, residents in better health tend to rely on their immune systems when facing health issues and therefore believe that community health service centers are sufficient for accessing medical care.

## 4. Discussion

### 4.1. Main Findings and Policy Implications

This study found that factors such as gender, age, and education level significantly affect residents’ willingness to participate in primary care initial diagnosis, responding to scholars’ calls for research [24,45,46,47]. The results showed that middle-aged people are more willing to prioritize primary care, whereas older people are less satisfied with the primary healthcare system and are unwilling to use primary care. This study hypothesized that the aging population prefers to go to high-level hospitals because, on the one hand, they favor convenience-based services, but community health service centers may not offer a convenient and fast visit. On the other hand, the elderly usually have more complex health conditions and trust the level of healthcare services in tertiary hospitals. According to the interview with R1, this phenomenon is explainable. It proves that a key reason for the elderly population’s reluctance to primary care initial diagnosis is that community health service centers do not provide the convenient access process they should. This is contrary to the policy objective of “guiding the elderly to primary healthcare institutions first” and hinders the implementation of the primary care initial diagnosis policy. Most previous studies concluded that highly educated people are more reluctant to choose primary care [24,48,49], and the results of this study showed that postgraduates with high education levels have the lowest willingness. This study suggests that highly educated people, who are more trusting and likely to have access to quality healthcare resources, directly choose high-level hospitals. This study argues that the highly educated population has more access to social resources and is more knowledgeable about hospital healthcare, which leads them to prefer high-level hospitals over community health centers. The interview record of R2 showed that the highly educated population prefers tertiary hospitals, which is consistent with questionnaire results. At the same time, it reveals a reason that the questionnaire failed to discover: that the highly educated population does not know enough about community health service centers. Therefore, the government and hospitals need to take different measures according to the characteristics of different populations. On the one hand, the government can help community health service centers promote publicity so that residents can understand the current situation, such as the medical standards of community hospitals, and enhance the confidence of the highly educated population in seeking medical treatment. On the other hand, the government can optimize the experience of the elderly in primary care, for example, by arranging volunteers to assist the elderly with their consultations and optimizing the consultation process for them. In this way, residents’ willingness to seek primary care will be enhanced.

Consistent with the research expectation and previous studies, most patients with common diseases prioritize community hospitals for consultation [50], and this phenomenon has largely alleviated the strain on medical resources in high-level hospitals, aligning with the requirements of the primary care initial diagnosis system and the results of the interviews. However, it is noteworthy that the majority of patients with chronic diseases are unwilling to receive primary care, which contradicts the goal of the primary care initial diagnosis policy [19]. Contrary to the findings of some scholars [51,52,53], this study concludes that most patients with chronic diseases still lack awareness of “prioritizing primary care for chronic diseases” and that the primary care initial diagnosis policy has not realized its potential advantages in managing chronic diseases. The interview transcripts of R4 and R5 confirmed the quantitative findings that the majority of patients with chronic diseases were reluctant to visit community healthcare service centers and had a low willingness to engage in primary care initial diagnosis. The interview transcripts also reveal that the shortage of medication and cumbersome consultation processes have largely deterred patients with chronic diseases from choosing primary care initial diagnosis. Policymakers need to be aware that the “doctor’s level of treatment” is an important factor affecting patients’ primary care. When guiding residents to primary care initial diagnosis, the government should, on the one hand, emphasize the crucial role of community health service centers and highlight it in policy revisions, appropriately allocate high-quality medical resources to these centers, strengthen general practitioners’ training, and increase the stockpile of medical drugs. Drawing on the medical experiences of developed countries such as the United Kingdom, the role of primary care doctors as gatekeepers should be utilized to ensure the orderliness of health care services. On the other hand, community health service centers need to focus on the development and use of technology to improve their level of care. With the help of the family doctor contracting mechanism and Internet technology, community health service centers should map out the status of patients with chronic diseases within their own communities and undertake tasks such as creating patient records, reminding them of follow-up consultations, and arranging for the preparation of medicines to provide better services.

The results of this study showed that a “level of confidence in the government” significantly affects residents’ willingness to seek primary care initial diagnosis. This study argues that government departments play a crucial role in formulating and implementing policies in the process of policy execution [29,30]. Government decisions greatly affect hospitals and the public. In the interview with R6, we learned that for the government to encourage public implementation of the primary care initial diagnosis policy, it must formulate sound policies and adopt reasonable policy measures. The government needs to build a better political image, enhance its credibility, and propose policy measures that are more conducive to the public. When the public has confidence in the government, they are more likely to consciously comply with the primary care initial diagnosis policy. At the same time, attention must be paid to the important impact of personal health status and health literacy. When residents have confidence in their health status and medical knowledge, they are more likely to prioritize community health service centers, as shown in the interviews with R7 and R8. Government departments and hospitals should focus on providing guidance and using publicity to enhance public health literacy, creating a long-term impact. As the results of this study show, health literacy significantly affects residents’ willingness to seek primary care initial diagnosis. Governments need to screen online health information to ensure the public has access to correct and useful information. At the same time, it should increase regulatory efforts to protect residents’ private information. Equally important as online publicity is the focus on developing Internet healthcare. As stated by interviewees R9 and R10, Internet healthcare is changing the medical treatment process, and the government and hospitals must cooperate in its development so that the public forms the awareness of “seeking treatment at lower-level hospitals for minor illnesses and at higher-level hospitals for major illnesses”, enabling the better implementation of the primary care initial diagnosis policy.

### 4.2. Limitations and Future Research

Based on the residents’ perspectives, this study explores the current situation and factors influencing the willingness of Beijing residents to seek primary care initial diagnosis. First, the research population of this study consists of permanent residents of Beijing, which still differs significantly from the situation in some other cities, despite the fact that Beijing is a pioneer in healthcare reform and can provide valuable insights for other cities. In the meantime, this study does not provide a detailed description of the effects of policy implementation. Future research can build on this study by expanding the sample to other cities or countries to explore differences between them, thereby improving the understanding and implementation of the primary care initial diagnosis system. Second, both questionnaire and interview methods were employed in this study, and the data obtained reflected more subjective perspectives of Beijing residents. Future research could develop more objective and accurate measurement tools to gather more data. Moreover, future research could focus on the interactions between the influencing factors and draw more conclusions. In the meantime, the sample size should be calculated and determined in advance, with more consideration given to statistical data for people aged 60 and above. Third, the data used in this study were cross-sectional, which hindered the exploration of causal relationships. Future research could adopt a longitudinal method, using a longer period of time for the same group of residents, as a way to test the effectiveness of the primary care initial diagnosis system and determine whether it is reasonable or not.

## 5. Conclusions

This study used a self-developed questionnaire to explore the attitudes of Beijing residents toward the primary care initial diagnosis system and the factors influencing their willingness. This study found that different residents showed different attitudes toward primary care initial diagnosis, specifically, residents aged 51–60 had the least willingness, the postgraduate population showed reduced willingness compared to others, and 97.7% of chronic disease patients were unwilling to receive primary care initial diagnosis. The government and hospitals need to take different measures according to different populations. In addition, there are many factors affecting residents’ willingness to seek primary care initial diagnosis, so the government and hospitals need to realize the importance of factors such as the level of confidence in the government, health literacy, the level of trust in Internet medical care, and take measures to improve the convenience of residents’ primary care initial diagnosis. This study innovatively introduces the influencing factors that have not been noticed in previous studies and provides suggestions for the implementation of the primary care initial diagnosis policy in China.

## Figures and Tables

**Table 1:** Basic information regarding residents.

Item	N	%
Gender		
Male	304	49.8
Female	306	50.2
Age		
20 years old and below	1	0.2
21 to 30 years old	147	24.1
31 to 40 years old	334	54.8
41 to 50 years old	121	19.8
51 to 60 years old	7	1.1
Education level		
High school and below	59	9.7
Junior college	115	18.9
Undergraduate	383	62.8
Postgraduate	53	8.7
Insurance type		
Basic medical insurance for urban employees	459	75.2
Basic medical insurance for urban residents	74	12.1
New rural co-operative medical insurance	37	6.1
Commercial health insurance	28	4.6
Public-funded medical service insurance	12	2.0
Occupation		
Peasantry	12	2.0
Employees of enterprises and public institutions	504	82.6
Self-employed	52	8.5
Emeritus and retired	1	0.2
Freelancer	29	4.8
Unemployed	12	2.0
Have a chronic disease		
Yes	128	21.0
No	482	79.0

Note: N represents the number of people, % represents the percentage.

**Table 2:** Residents’ choice of hospital level according to the type of disease.

Disease Type	Community Health Service Center		Primary Hospital		Secondary Hospital		Tertiary Hospital	
	N	%	N	%	N	%	N	%
Common disease	493	80.8	94	15.4	13	2.1	10	1.6
Acute disease	2	0.3	144	23.6	274	44.9	190	31.1
Chronic disease	14	2.3	134	22.0	405	66.4	57	9.3
Severe disease	0	0	2	0.3	96	15.7	512	83.9
Difficult miscellaneous disease	2	0.3	9	1.5	97	15.9	502	82.3
Recover after illness	187	30.7	247	40.5	149	24.4	27	4.4

Note: N represents the number of people, % represents the percentage.

**Table 3:** Univariate analysis of residents’ willingness regarding primary care initial diagnosis.

Item	Mean	SD	F	*p*
Gender			33.101	0.000
Male	4.48	0.703		
Female	4.12	0.820		
Age			5.174	0.000
20 years old and below	4.00	-		
21 to 30 years old	4.10	0.894		
31 to 40 years old	4.35	0.743		
41 to 50 years old	4.45	0.695		
51 to 60 years old	3.71	0.756		
Education level			8.797	0.000
High school and below	4.37	0.786		
Junior college	4.29	0.825		
Undergraduate	4.37	0.739		
Postgraduate	3.79	0.840		
Insurance type			1.163	0.326
Basic medical insurance for urban employees	4.31	0.780		
Basic medical insurance for urban residents	4.36	0.823		
New rural co-operative medical insurance	4.30	0.661		
Commercial health insurance	4.07	0.766		
Public-funded medical service insurance	4.00	1.044		
Occupation			0.985	0.426
Peasantry	4.50	0.798		
Employees of enterprises and public institutions	4.32	0.772		
Self-employed	4.21	0.800		
Emeritus and retired	4.00	-		
Freelancer	4.24	0.830		
Unemployed	3.92	1.084		
Have a chronic disease			0.467	0.495
Yes	4.34	0.818		
No	4.29	0.776		

Note: SD represents standard error; *p* < 0.05 represents statistical significance.

**Table 4:** Multiple linear regression analysis of residents’ willingness regarding primary care initial diagnosis.

Influencing Factors	Mean	Beta	SD	S Beta	t	*p*	95% Confidence Interval for Beta	
							Upper Limit	Lower Limit
(Constant)		−1.053	0.377		−2.790	0.000	−1.794	−0.312
State of an illness	4.577	−0.048	0.044	−0.034	−1.095	0.274	−0.133	0.038
Level of confidence in the government	4.248	0.320	0.037	0.316	8.570	0.000	0.246	0.393
Satisfaction with previous health reform policies	4.423	−0.008	0.039	−0.007	−0.213	0.832	−0.086	0.069
Personal health status	4.074	0.276	0.037	0.265	7.462	0.000	0.203	0.349
Health literacy	3.254	0.468	0.115	0.204	4.065	0.000	0.242	0.694
E-Health literacy	4.313	−0.247	0.112	−0.161	−2.206	0.028	−0.466	−0.027
Level of trust in Internet medical care	3.951	0.587	0.206	0.280	2.851	0.005	0.183	0.992

Note: SD represents standard error, Beta represents unstandardized coefficient, S Beta represents standardized coefficient; *p* < 0.05 represents statistical significance.

**Table 5:** Residents’ views on primary care initial diagnosis.

Interviewee	Gender	Age	Interview Transcripts
R 1	Female	70	Occasionally, I opt for medical services at a community health service center, but I must admit that some of my experiences have been unsatisfactory. Despite the center’s modest size, I did not find it very convenient to seek treatment, which sometimes deters me from choosing it as my primary option.
R 2	Male	32	Personally, I’m aware of the primary care initial diagnosis policy and I’m willing to implement it. However, I don’t know enough about the health service center in my community, so sometimes I would rather go to a tertiary hospital, even though it is more troublesome.
R 3	Male	36	In my opinion, with the implementation of the primary care initial diagnosis policy, patients with common diseases are more willing to choose the nearest community health service centers for treatment.
R 4	Female	52	I have been suffering from a chronic illness and have been prescribed drugs and treatment at a tertiary hospital for the last few years, which is a more regular form of medical treatment for me. There are two community health centers near me, but they don’t have the drugs I need, so I have to go to the tertiary hospital.
R 5	Male	66	I have sought treatment at both community health service centers and tertiary hospitals. For chronic conditions, registering and explaining my situation to a new doctor each time can be cumbersome. I haven’t noticed many advantages at community health centers in managing chronic diseases. If there were clear benefits compared to tertiary hospitals, I would prefer to choose it.
R 6	Male	37	For me, the government healthcare sector is the main player in promoting the implementation of policies. Therefore, I think that the government sector must give people hope and instill confidence in the residents, so that they will believe in the power of the primary care initial diagnosis policy.
R 7	Male	59	Although I’m older, I usually keep up with my exercise, so my health is in good shape. When I have a minor issue such as a cold or fever, I believe in my body’s ability to recover and feel that the community health service centers can provide me with the treatment I need.
R 8	Male	30	I usually get health information from the Internet to learn about diseases and treatments. When someone in my family gets sick, I make the first judgment call and can buy the medication myself for treatment.
R 9	Female	45	The rapid development of the Internet has also accelerated the growth of the medical industry. I used to have to go to the hospital for medical treatment, but now I can diagnose through the Internet and then pick up the medication offline, which has brought great convenience to the patients.
R 10	Female	40	Internet healthcare has not only resulted in fewer bills for patients, but in some cases, patients can even receive treatment without leaving their homes. This not only saves time but also reduces traveling expenses.

Note: R represents resident.

## Data Availability

The datasets during and/or analyzed during the current study are available from the corresponding author upon reasonable request.
